# ﻿A preliminary review of *Isonychia* Eaton, 1871 from Chinese mainland with a re-description of *I.kiangsinensis* Hsu, 1936 (Insecta, Ephemeroptera, Isonychiidae)

**DOI:** 10.3897/zookeys.1178.104619

**Published:** 2023-09-04

**Authors:** Xin-He Qiang, Chang-Fa Zhou

**Affiliations:** 1 College of Life Sciences, Nanjing Normal University, Nanjing 210023, China Nanjing Normal University Nanjing China

**Keywords:** Biogeography, mayfly, morphology, species diversity, taxonomy

## Abstract

Previously, seven species of the genus *Isonychia* Eaton, 1871 were reported in China, but they have never been systematically reviewed. After examining our collections from the Chinese mainland, six species and one additional subspecies have been recognized, compared, and photographed. Among them, *I.kiangsinensis* is redescribed in all stages and a neotype is designated. Its males have triangular penes and nymphs have three dark pigments on each gill. A synonym of *I.guixiensis*Wu et al., 1992 (*I.sinensis*Wu et al., 1992) is confirmed. The males of this species have nearly cylindrical penes and clear abdominal markings. Finally, two species and one subspecies are recorded for the first time in China: *I.ussuricasibirica*Tiunova et al., 2004, *I.ussuricaussurica* Bajkova, 1970 and *I.vshivkovaevshivkovae*Tiunova et al., 2004. Together with the *I.ignota* (Walker, 1853), *I.sexpetala*Tiunova et al., 2004, *I.formosana* (Ulmer, 1912) and possible *I.japonica* (Ulmer, 1920), they show the rich diversity of the genus *Isonychia* in China.

## ﻿Introduction

The mayflies of the genus *Isonychia* are relatively large and common aquatic insects. They can be found in almost all of our sampling sites in China and are among the earliest reported species of Chinese Ephemeroptera. For example, *I.formosana* (Ulmer, 1912) was named based on materials from Taiwan province. However, since then, although a series of researchers have reported and described six additional *Isonychia* species from Chinese mainland (Ulmer 1925; Hsu 1936; She and You 1988; Wu et al. 1992; Tiunova et al. 2004), no comprehensive review has been conducted.

Hsu (1936) named the species *I.kiangsinensis* based on male imagos and subimagos. Fifty years later, You and Su (1987) described its nymphal stage. However, their original descriptions were extremely brief, their drawings were not accurate, and the types of this species were lost, making the exact morphology and taxonomic status of this species unclear.

Similarly, three other species of Chinese *Isonychia* have been reported based on imagoes only. She and You (1988) described *I.hainanensis* from Hainan province, southern China. Wu et al. (1992) named two additional species (*I.sinensis*Wu et al., 1992and *I.guixiensis*Wu et al., 1992) from Jiangxi province, central China. All of these species need to be compared in detail and their exact characters need to be illustrated.

Furthermore, we believe that there is some confusion in the historical studies on Chinese *Isonychia*. For instance, 1) the penes in the original picture of *I.guixiensis* are much smaller than those of others and look like the membranous processes of other congeners. 2) Tiunova et al. (2004) synonymized the species *I.hainanensis* She & You, 1988 with *I.ignota* (Walker, 1853) without checking any Chinese materials or types. 3) Ulmer (1925) reported *I.japonica* (Ulmer, 1920) (as *Chirotonetesjaponicus*) from China, but Tiunova et al. (2004) confirmed that this species is found in Japan only and doubted the report of Ulmer (1925).

To solve these issues mentioned, it is necessary to systematically revise Chinese *Isonychia* specimens, especially those from northeastern China. In recent years, we have thoroughly checked our mayfly collections of the family Isonychiidae, focusing on the known species. As a preliminary result, we confirmed six species and one additional subspecies, including three new records, while leaving several possible new species and *I.formosana* for future studies. The main taxonomic characters of these species are photographed and presented to benefit future works.

## ﻿Materials and methods

The species concept and delimitation are based on morphology, and species differentiation mainly follows the work of Tiunova et al. (2004).

The nymphs were collected by hand net, and the adults were attracted by lights. Some of the adults were reared indoors from mature nymphs (mature nymphs were put into a plastic tray with some water from the creeks they lived. The whole system was covered with a mosquito net and oxygen was supplied with a small fish pump). All materials were stored in ethanol (higher than 80%).

All specimens were examined under a stereo microscope (Mingmei Photoelectric, MZ81, Guangzhou, China) and photographed with a digital camera (Single Lens Reflex, Guangzhou, China). Some digital photos of whole nymphs and adults were taken by Sony a7R (Interchangeable Lens Digital Camera). Some small structures such as mouthparts, claws, and penes, were observed and photographed under a microscope camera (Nikon Eclipse 50i, Tokyo, Japan). Eggs were dissected from female imagos, fixed in 4% glutaraldehyde for 5–8 hours to preserve their structure, dehydrated using a series of ethanol solutions (30%, 50%, 70%, 90%, and 100%, 10–15 min each), critically dried to prevent artifacts, mounted onto stubs, coated with gold film in a vacuum, and photographed with a scanning electron microscope (Apreo 2S, Thermo Fisher Scientific Company, Massachusetts, USA). The distribution map is downloaded from the website (http://bzdt.ch.mnr.gov.cn/). All specimens used in this study are deposited in the Mayfly Collection, College of Life Sciences, Nanjing Normal University (**NNU**), China.

## ﻿Taxonomic account

### ﻿ Isonychia guixiensis

Taxon classificationAnimaliaEphemeropteraIsonychiidae

Wu et al., 1992

2A942885-FB09-5C21-BF35-2BDA6808203E

Isonychia guixiensis Wu et al., 1992: 78 (male, female). Type from Jiangxi province, China; You and Gui 1995: 25 (male); Gui and Zhou 1999: 126.
Isonychia sinensis Wu et al., 1992: 79 (male, female). Type from Jiangxi province, China; You and Gui 1995: 27 (male); Su and Zhu 1997: 121 (egg) (synonymized by Zhou 2013: 196).

#### Material examined.

***Holotype*** of *I.guixiensis* (male imago), Guixi City, Jiangxi Province, China, 4–6-VI-1990, collected by Lixin Tian, Lianfang Yang; paratypes of *I.guixiensis*: 10 male imagos and 12 female imagos, same data as the holotype. ***Holotype*** of *I.sinensis* (male imago), Guixi City, Jiangxi Province, China, 4–6-VI-1990, collected by Lixin Tian, Lianfang Yang; paratypes of *I.sinensis*: 5 male imagos and 13 female imagos, same data as the holotype.

#### Diagnosis.

**Figure 1.**
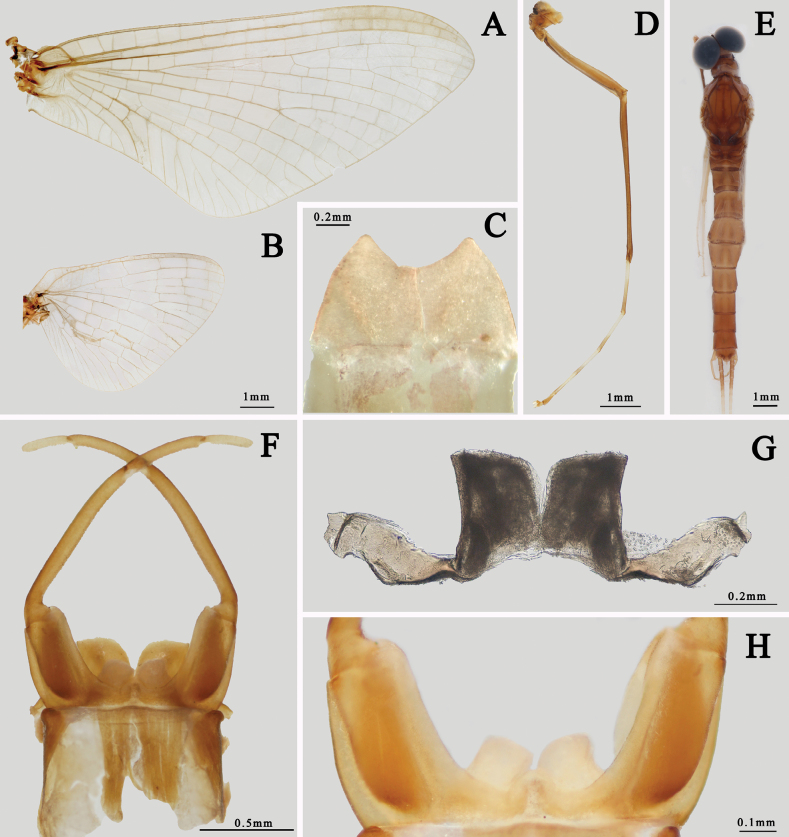
Imaginal structures of *I.guixiensis***A** forewing of male **B** hindwing of male **C** subanal plate of female (ventral view) **D** foreleg of male **E** abdomen of male (dorsal view) **F** male genitalia (ventral view) **G** penes (dorsal view) **H** membranous processes (ventral view).

The male of *I.guixiensis* has transparent wings (Fig. 1A, B), dark brown forefemora, foretibiae, apical half of tarsal segment I–IV and tarsal segment V (Fig. 1D). Its abdominal terga are brown with a pale median line and dark longitudinal submedian stripes (Fig. 1E). The penes are nearly cylindrical with pointed apicolateral angles, and membranous processes beneath penes are clear (Fig. 1F–H). The females are similar to the males, with concave sternum IX (Fig. 1C).

This species and *I.kiangsinensis* can be found sympatrically in Jiangxi province, central China. They can be differentiated by the following characters: (1) *I.guixiensis* is smaller than *I.kiangsinensis*; (2) the body of *I.guixiensis* is brown to dark brown (Fig. 1E) while the body of *I.kiangsinensis* is usually pink to reddish; (3) *I.guixiensis* has distinct brown markings on abdomen (Fig. 1E) while the *I.kiangsinensis* has none. (4) their penes are dissimilar: those of *I.kiangsinensis* are triangular but penes of *I.guixiensis* are nearly cylindrical with pointed apicolateral angles (Fig. 1G).

#### Description.

See Wu et al. (1992).

#### Distribution.

China (Jiangxi province).

#### Remarks.

Wu et al. (1992) named two species, *I.guixiensis and I.sinensis*, in a single paper, with the penes of *I.guixiensis* appearing remarkably small. After checking all types of those two species, we found no difference between them, and their abdominal markings are absolutely less distinct than those in the original figures of Wu et al. (1992), and the synonymy of two species is confirmed here. Because the name of *I.guixiensis* is before *I.sinensis* in the same paper, the former is retained as the valid name.

### ﻿ Isonychia ignota

Taxon classificationAnimaliaEphemeropteraIsonychiidae

(Walker, 1853)

CB5D5418-18E3-5B1E-926A-3425FA28E1BC

Baetis ignotus Walker, 1853: 571 (type locality: unknown, probably western Europe; holotype, male, in Natural History Museum, London; figured by Kimmins 1960: 275).
Isonychia ignota : Eaton 1871: 135.
Isonychia ferruginea Albarda, 1878: 128 (synonymized by Eaton 1885: 205).
Siphlurus sp.: Rostock 1878: 88 (synonymized by Eaton 1885: 205).
Chirotonetes ignotus : Eaton 1885: 205.
Palingenia roeselii Joly, 1871: 3 (adults and nymph) (transferred to Jolia by Eaton 1881: 192) (synonymized by Needham 1905: 28).
Isonychia (Isonychia) ignota : Kondratieff and Voshell 1983: 134; Tiunova et al. 2004: 7 (nymph and adults).
Isonychia hainanensis She et You, 1988: 29 (adults) (synonymized by Tiunova et al. 2004: 7).

#### Material examined.

3 male imagos, 9 female imagos, 8 male subimagos, 2 female subimagos, and 15 nymphs, Bawangling, Hainan Province, China, 14-IX-2015, collected by Qin Si, Junzhi Sun, Juanyan Luo.

#### Diagnosis.

**Figure 2.**
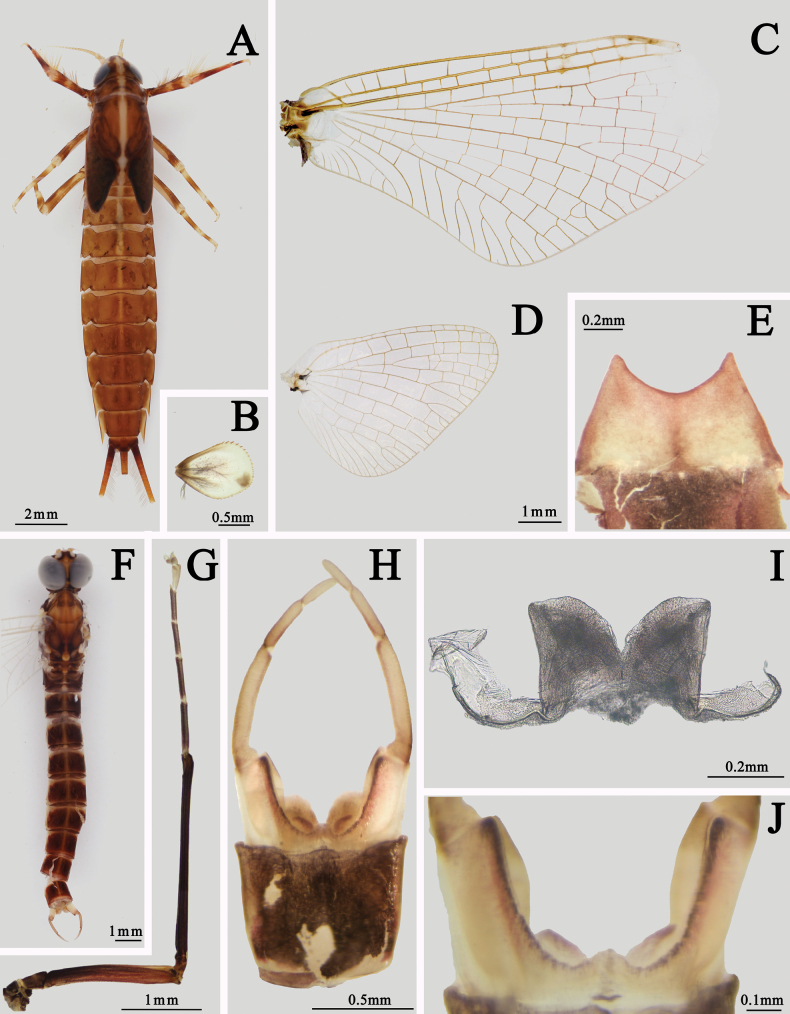
Imaginal and nymphal structures of *I.ignota***A** nymph (dorsal view) **B** gill VII **C** forewing of male **D** hindwing of male **E** subanal plate of female (ventral view) **F** body of male imago (dorsal view) **G** foreleg of male **H** male genitalia (ventral view) **I** penes (dorsal view) **J** membranous processes (ventral view).

The nymph of this species can be identified by its body with a pale midline from head to abdominal terga VII, tergum X with dark posterior half, and its gills with spines along whole margins (Fig. 2A, B). The male imago is characterized by its wings without any markable painting or pigments, MP of hindwing forked more apically than MA, and two forking points are distinct (Fig. 2C, D), almost total dark forelegs, especially its foretarsi (apical segment usually darker) (Fig. 2G), penes with clear membranous processes ventrally, and apical margin of penis distinctly convex (Fig. 2H–J). The body color is almost brownish to dark brown, with a clear median, longitudinal, pale line on terga I–X, anterior margins of each tergum pale (Fig. 2F). Female imago has a similar color pattern of body and foreleg to the male. Veins of wings are reddish brown to dark, very clear. Posterior margin of sternum IX is shallowly concave (Fig. 2E).

Generally, the males of this species can be identified by their uniform dark forelegs and shorter segment II of gonostyli. The nymphs can be classified by their gills (color and spine pattern) and color pattern of their abdomen.

#### Description.

See Tiunova et al. (2004).

#### Distribution.

China (Hainan province); Mongolia; Russia; Western Europe (Tiunova et al. 2004).

#### Remarks.

This species was first recorded in China as *I.hainanensis* by She and You (1988). However, Tiunova et al. (2004) synonymized it with the species *I.ignota*. We checked the holotype of the former species and found only very delicate and slight differences between them, like color and shape of penes, but we believe those differences are caused by long-term storage in alcohol of the holotype. In April 2023, we also visited some localities of the types in Hainan Island, China, and collected many specimens of the genus *Isonychia*. However, we did not find any of *I.hainanensis*. Therefore, we think this synonym is correct. Our record shows the species *I.ignota* has a wide distribution, from western Europe to southern China.

### ﻿ Isonychia kiangsinensis

Taxon classificationAnimaliaEphemeropteraIsonychiidae

Hsu, 1936

80825DE5-1957-5897-B2EF-85F0915BF079

Isonychia kiangsinensis Hsu, 1936: 323 (male, male subimago). Types from Shang Jao, Sheng Mi, Jiangxi province, China.
Isonychia kiangsinensis : Gui 1985: 80; You and Su 1987: 334 (nymph); You and Gui 1995: 23; Su and Zhou 1998: 28; Gui et al. 1999: 326; Zhou et al. 2015: 118 (adult, nymph); Tiunova et al. 2004: 2; Vasanth et al. 2019: 169; Muthukatturaja et al. 2021: 284).

#### Material examined.

Designated neotype (male imago), Leiguling Water, Wuyishan Nature Reserve, Jiangxi Province, China, 27°99142′N, 117°89111′E, 424 m, 4-VI-2005, collected by Lianfang Yang, Christy Jo Geraci. 80 male imagos, same data as the neotype; 1 male imago, 2 female imagos, 1 male subimago, 4 female subimagos, 1 nymph, and 7 exuviae of nymphs, grass carp Pond, Jingning She Autonomous County, Lishui City, Zhejiang Province, China, 11–12-VIII-2020, collected by Xuhongyi Zheng, Zhenxing Ma; 1 male imago, 5 female imagos, and 2 male subimagos, Dagu Mountain Scenic Area, Yi County, Huangshan City, Anhui Province, China, 4-X-2021, collected by Xuhongyi Zheng, Dewen Gong; 7 nymphs and 3 exuviae of nymphs, Nanping City, Fujian Province, China, 118°7′38″E, 26°38′12″N, 190 m, 4-V-2021, collected by Zhengxin Ma, Xuhongyi Zheng; 16 male imagos and 20 female imagos, Daqiutan, Jiulianshan Forest Farm, Longnan County, Jiangxi Province, China, 10-XII-2005, collected by Changfa Zhou, Changhai Sun.

#### Diagnosis.

**Figure 3.**
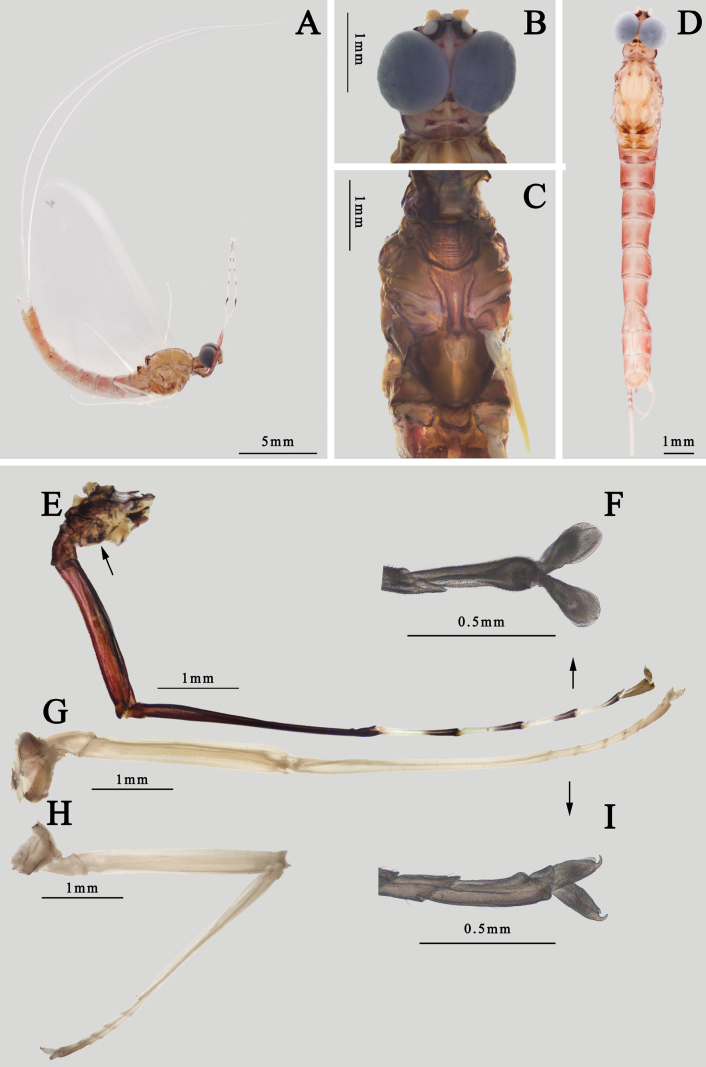
Structures of *I.kiangsinensis* male imago **A** habitus (lateral view) **B** head (dorsal view) **C** thorax (ventral view) **D** abdomen (dorsal view) **E** foreleg (arrow indicating the gill relics on forecoxa) **F** claw of foreleg **G** midleg **H** hindleg **I** claw of midleg.

**Figure 4.**
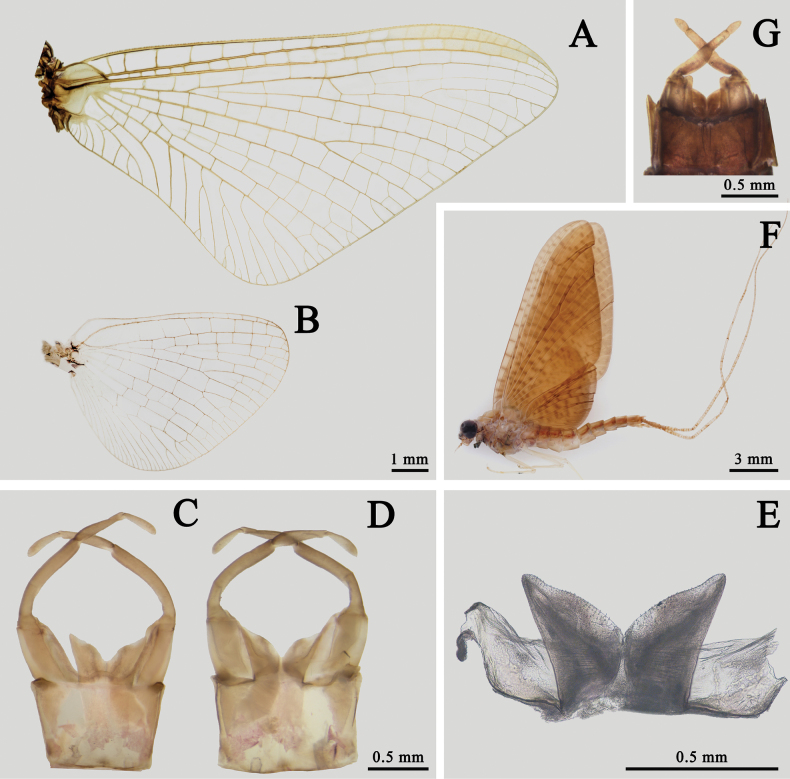
Male structures of *I.kiangsinensis***A–E** male imago **F–G** male subimago **A** forewing **B** hindwing **C** male genitalia (ventral view) **D** male genitalia (dorsal view) **E** penes (ventral view) **F** habitus (lateral view) **G** genitalia (ventral view).

The male imago of the species *I.kiangsinensis* can be identified by following characters: (1) the body is almost pink to reddish brown (Fig. 3A, D). (2) wings totally transparent except semitransparent pterostigma, without any other markable painting or pigments (Fig. 4A, B). (3) each penis near triangular with oblique apical margin (Fig. 4E); (4) inner margin of segment II of gonostylus slightly concave or straight; the combined length of segments III and IV subequal to segment II (Fig. 4C, D). (5) forefemora and foretibiae totally dark brown; foretarsal segments I–IV pale in basal half and dark in apical half, segment V gray to dark (Fig. 3E); (6) foretarsi are longer than tibiae, their length ratio is 1.1: 1.0 (Fig. 3E).

In contrast to *I.ussuricaussurica* Bajkova, 1970, *I.ussuricasibirica*Tiunova et al., 2004, and *I.vshivkovaevshivkovae*Tiunova et al., 2004 (see below), *I.radhae*Muthukatturaja et al., 2021, forewings of *I.kiangsinensis* are transparent, without clear markings (Fig. 4A, B). Unlike *I.guixiensis*, *I.ignota*, and *I.moyarensis*Vasanth et al., 2019, abdomen of *I.kiangsinensis* is pink to reddish, with a distinct median pale line but without any obvious dark markings (Fig. 3D). Although *I.kiangsinensis* and *I.ignota* have similar transparent wings and gonostyli (segment II is relatively short), their penes and forelegs are different: (1) *I.kiangsinensis* has triangular penes (Fig. 4C–E), but those of *I.ignota* are nearly cylindrical with convex apical margins; (2) foretarsi of *I.kiangsinensis* have pale basal 1/2 and dark apical 1/2 (Fig. 3E), but those of *I.ignota* are totally dark; (3) foretarsi of *I.kiangsinensis* are longer than foretibiae while foretarsi of *I.ignota* are subequal to or shorter than foretibiae.

Four characters can be used to separate nymphs of *I.kiangsinensis* from other species: (1) abdominal terga I–X with clear pale median longitudinal line (Fig. 7A, C), (2) each gill lobe with two to three spots, the largest one in the middle dark purple, (3) gill lobes VI and VII with spines on apical margins (Fig. 10F, G). (4) Tergum X pale in anterior 1/2, brown in posterior 1/2 (Fig. 10J). Compared to known nymphs of Asian *Isonychia* species, gills of *I.kiangsinensis* usually have three markings, a large median purple one and two small brown ones. This character is somewhat unique. Generally, this species is larger, more reddish than others, and is distributed south of Yangtze River, China.

#### Description.

Also see Hsu (1936) or You and Su (1987). ***Male imago*** (in alcohol, Figs 3, 4): body length 15.0–16.0 mm, cerci 33.0–35.0 mm, forewings 14.0–15.0 mm, hindwings 7.0–8.0 mm. Body generally pink to reddish brown, head, and thorax slightly deeper than abdomen (Fig. 3A–D). Head: compound eyes almost covered all head, with dark 1/3 lower portion and grey to dark 2/3 upper portion; two eyes contiguous or near contiguous, only a very narrow suture between them. Ocelli with dark basal band, upper portion pale. Antennae pale, ventral surface of scape and pedicle pigmented with brown dots or irregular markings. Anterior margin of head nearly straight (Fig. 3B). Thorax: pronotum and anterior 1/3 mesonotum with clear dark brown longitudinal stripes but midline pale, median 1/3 mesonotum with a pair of dark brown oblique stripes sub-medially, mesoscutellum and metascutellum dark, other parts reddish brown to brown, including sterna. Basisternum of mesothorax narrowed progressively from anterior to posterior, apex bluntly expanded (Fig. 3C).

Wings totally transparent except semi-hyaline pterostigma, crossveins of all wings clear (Fig. 4A, B). Veins of forewings yellowish to reddish, MA forked at apical 1/3, MP forked slightly baser than Rs, asymmetrical; four pairs of forked and two unforked intercalaries between CuA and CuP, A connecting to hind margin with two veinlets (Fig. 4A). Hindwings with very shallow and blunt costal process at base. MA and MP forked equally, both of them forked symmetrical (Fig. 4B). Forelegs with deep reddish or chocolate forefemora and tibiae, apical 1/2 of each tarsus except segment V dark but basal 1/2 pale, segment V of foretarsi pale to grey (Fig. 3E); gill relics of forecoxa gray to dark (Fig. 3E); two claws similar, blunt, and plate-like (Fig. 3F). Midleg and hindleg pale, claws similar too but both of them acute (Fig. 3G–I). Length ratio of forefemora: tibiae: tarsi = 1.0: 1.4: 1.6, length ratio of foretarsal segments from I to V = 1.0: 0.8: 0.6: 0.5: 0.4; length ratio of mid-femora: tibiae: tarsi = 1.0: 1.3: 0.6, length ratio of mid-tarsal segments from I to V = 1.0: 1.2: 0.8: 0.8: 1.4; length ratio of hind femora: tibiae: tarsi = 1.0: 0.9: 0.5, length ratio of hind-tarsal segments from I to V = 1.0: 0.9: 0.7: 0.5: 1.2. Abdomen: terga reddish brown to pink, with a median longitudinal pale line, posterior margins of each tergum brown to dark, deeper than other parts. Base of cerci reddish brown, other part pale, surface with tiny setae. Terminal filament pale to reddish brown, six or seven segments (Fig. 3D). Genitalia: subgenital plate deeply incaved with a semi-circular median lobe; inner surface of second segment of gonostylus concave; length ratio of four segments of gonostylus from base to apex = 1.0: 7.0: 3.5: 2.5 (Fig. 4C, D). Two penes fused at basal 1/3 but bifurcated at apical 2/3, slightly bent laterally; each penis near triangular with a sharp apex (Fig. 4E).

***Male subimago*** (in alcohol, Fig. 4F, G): body length 12.0–13.0 mm, cerci 19.0–20.0 mm. Length ratio of forefemora: tibiae: tarsi = 1.0: 1.1: 1.0, length ratio of foretarsal segments I: II: III: IV: V = 1.0: 0.6: 0.6: 0.6: 0.8; length ratio of mid-femora: tibiae: tarsi = 1.0: 0.8: 0.4, length ratio of mid-tarsal segments I: II: III: IV: V = 1.0: 0.7: 0.8: 0.4: 1.0; length ratio of hind femora: tibiae: tarsi = 1.0: 0.6: 0.5, length ratio of hind-tarsal segments I: II: III: IV: V = 1.0: 0.8: 0.5: 0.6: 1.5. Body duller than male imago, wings semi-hyaline, amber to brown. Crossveins of wings surrounded with grey to dark cloud (Fig. 4F). Genitalia generally similar to those of male imago but apical margin of penes only slightly oblique, second segment of gonostylus thickened (Fig. 4G).

**Figure 5.**
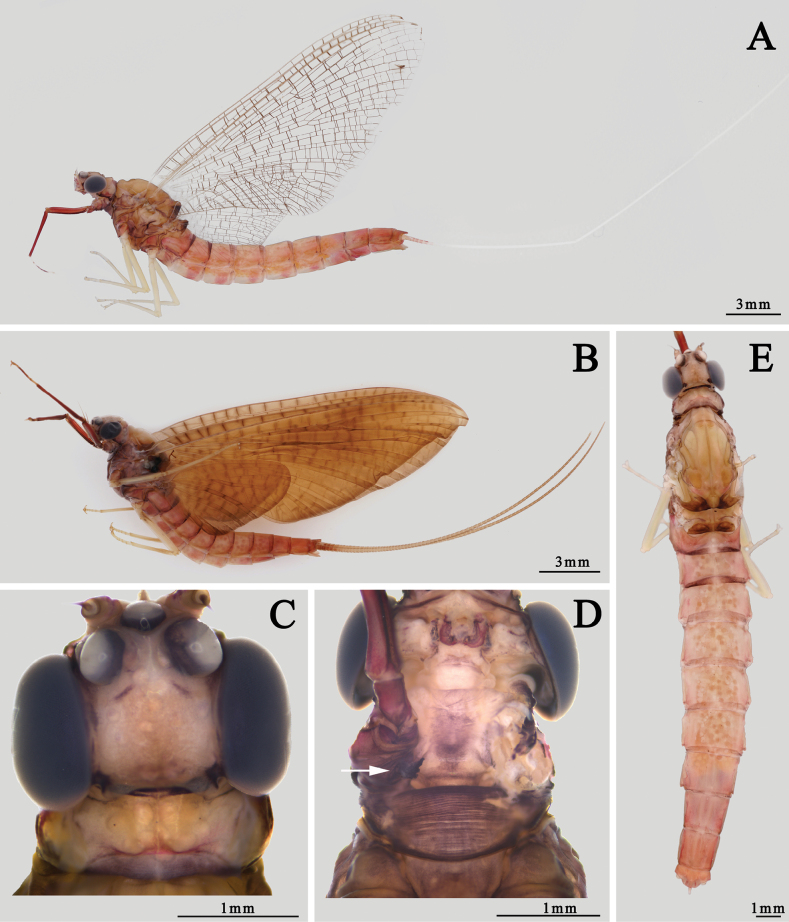
Female structures of *I.kiangsinensis***A** imago (lateral view) **B** subimago (lateral view) **C** head of imago (dorsal view) **D** head and prothorax of imago (ventral view, arrow indicating the gill relics on forecoxa) **E** abdomen of imago (dorsal view).

**Figure 6.**
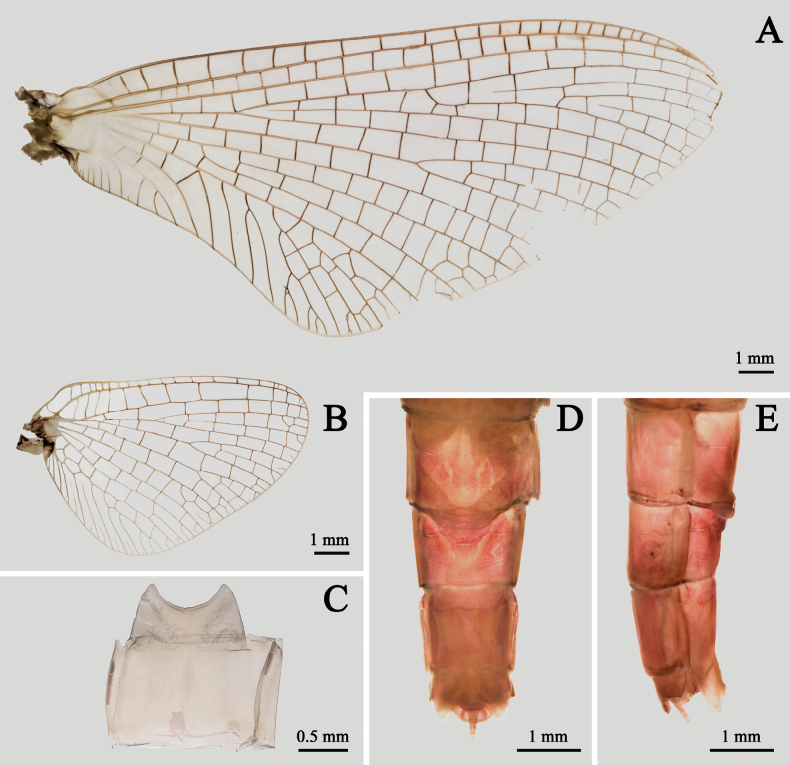
Structures of *I.kiangsinensis* female imago **A** forewing **B** hindwing **C** subanal plate **D** abdominal segments VII–X (ventral view) **E** abdominal segments VII–X (lateral view).

***Female imago*** (in alcohol, Figs 5, 6): body length 20.0–21.0 mm, cerci 42.0–44.0 mm, forewings 19.0–20.0 mm, hindwings 7.8–8.8 mm. Length ratio of forefemora: tibiae: tarsi = 1.0: 1.5: 1.6, and length ratio of foretarsal segments from I to V = 1.0: 0.9: 0.8: 0.5: 0.8; length ratio of mid-femora: tibiae: tarsi = 1.0: 1.1: 0.5, and length ratio of mid-tarsal segments from I to V = 1.0: 0.9: 0.6: 0.6: 1.5; length ratio of hind femora: tibiae: tarsi = 1.0: 0.8: 0.5, and length ratio of hind-tarsal segments from I to V = 1.0: 0.9: 0.5: 0.5: 1.5. Body color pattern similar to male but paler (Fig. 5A). Compound eyes dark, distance between them ~ 3× diameter of ocellus. Two dark dots on occiput nearby eyes (Fig. 5C). Gill relics of forecoxa gray to dark (Fig. 5D). Veins of wings clearer than males, MP of hindwing forked more apically than MA (Fig. 6A, B). Abdomen more reddish than male, posterior 1/2 of tergum and sternum usually deeper than anterior 1/2 (Fig. 5E). Sternum VII extended posteriorly into a small lobe (Fig. 6D, E), sternum IX narrowed progressively and smoothly from base, posterior margin incaved into semi-circular shape (Fig. 6C).

***Female subimago*** (in alcohol, Fig. 5B): body length 14.0–15.0 mm, cerci 17.0–18.0 mm. Length ratio of forefemora: tibiae: tarsi = 1.0: 1.3: 0.6, length ratio of foretarsal segments I: II: III: IV: V = 1.0: 0.6: 0.7: 0.4: 0.8; length ratio of mid femora: tibiae: tarsi = 1.0: 0.7: 0.4, length ratio of mid-tarsal segments I: II: III: IV: V = 1.0: 0.8: 0.6: 0.4: 1.2; length ratio of hind femora: tibiae: tarsi = 1.0: 0.7: 0.3, length ratio of hind-tarsal segments I: II: III: IV: V = 1.0: 0.7: 0.6: 0.5: 1.5. Similar to female imago but body duller and wings semi-hyaline, amber to brown.

**Figure 7.**
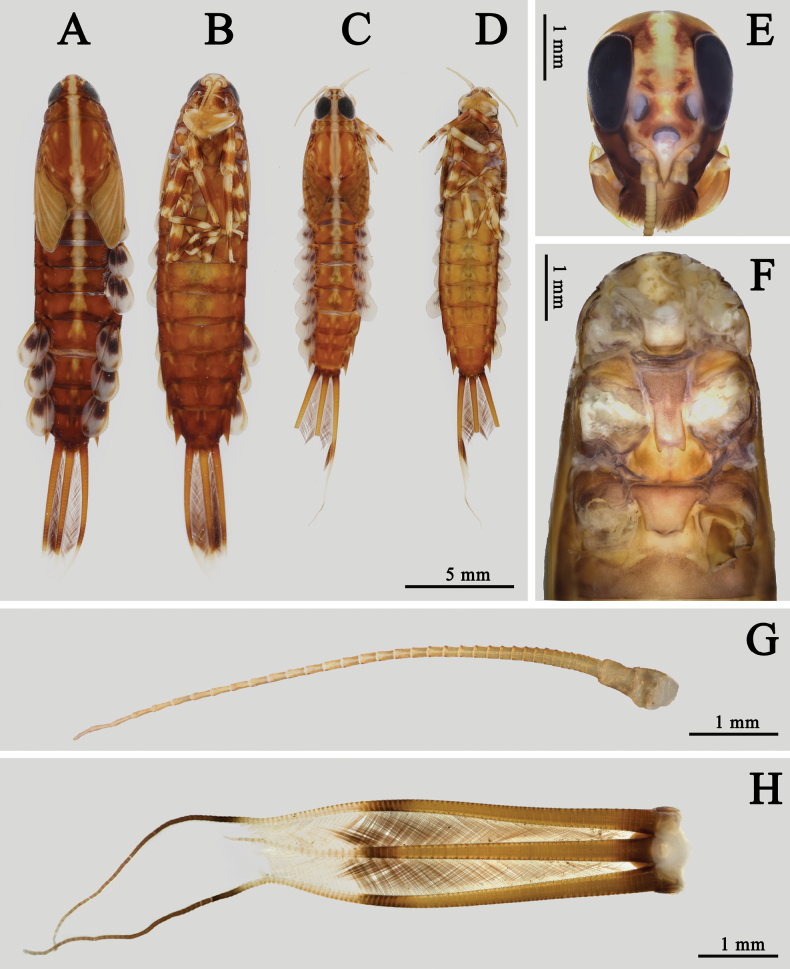
Nymphal structures of *I.kiangsinensis***A** female nymph (dorsal view) **B** female nymph (ventral view) **C** male nymph (dorsal view) **D** male nymph (ventral view) **E** head of female **F** thorax of female (ventral view) **G** antenna **H** caudal filaments.

**Figure 8.**
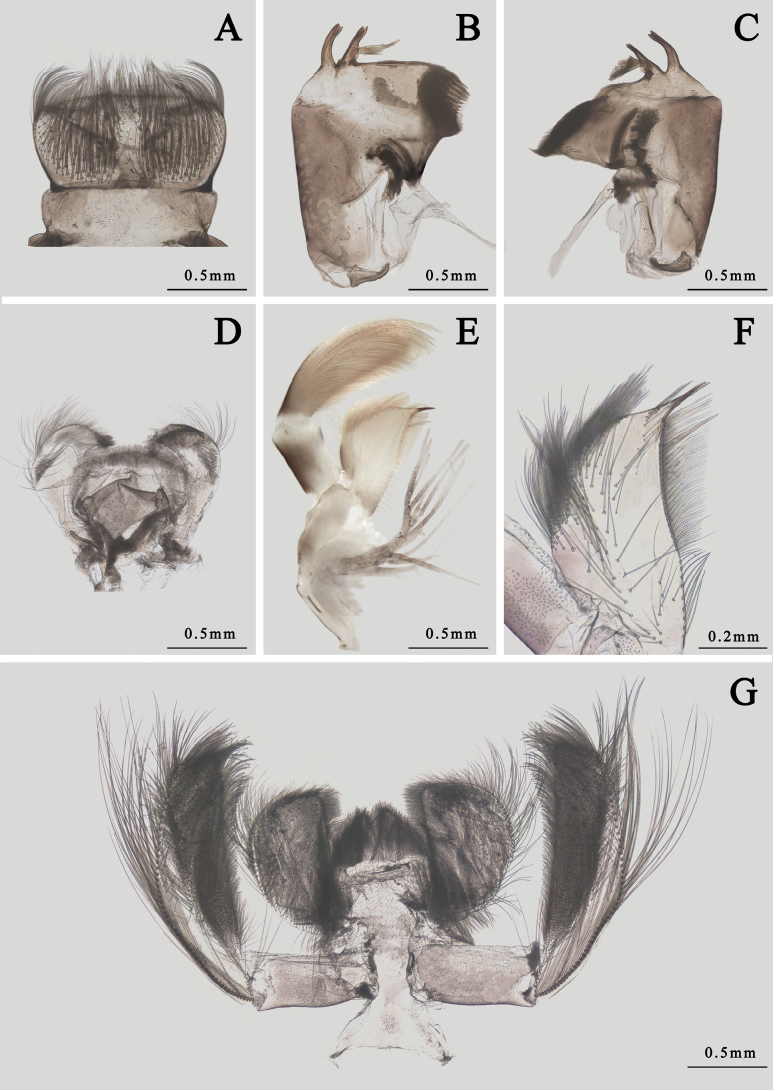
Mouthparts of *I.kiangsinensis* nymph **A** labrum **B** left mandible **C** right mandible **D** hypopharynx **E** maxilla **F** galea-lacinia of maxilla **G** labium.

**Figure 9.**
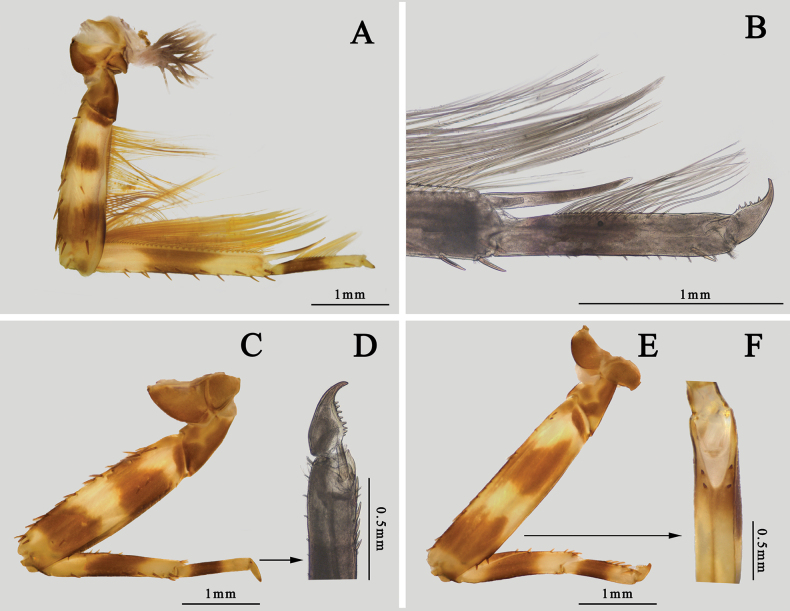
Nymphal legs of *I.kiangsinensis***A** foreleg **B** apical spine of foretibiae **C** midleg **D** claw of midleg **E** hind leg **F** ventral cleft of hind femur (ventral view).

**Figure 10.**
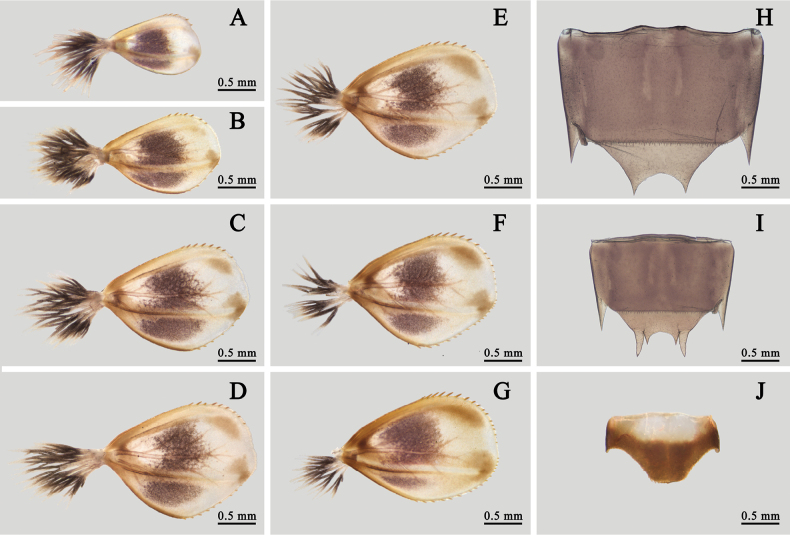
Nymphal structures of *I.kiangsinensis***A**–**G** gills I–VII **H** female sternite IX (ventral view) **I** male sternite IX (ventral view) **J** tergite X (dorsal view).

***Nymph*** (in alcohol, Figs 7–10): body length 13.0–17.0 mm, cerci 8.0–10.0 mm, terminal filament 5.0–6.5 mm; body brown to dark amber, with a clear pale median longitudinal line; legs and tail with pale and dark bands, gills with purple markings (Fig. 7A–D). Head: genae brown, lateral 1/3 clypeus brown but median 1/3 pale; scape and pedicel of antennae darker than others, antennae smooth. Length of antennae ~ 3× width of head. Frontal carina pale, area between three ocelli brown, midline of head pale, area between median ocellus and compound eyes pale too. Frontal carina between two antennae, very sharp. Vertex smooth and convex. Dark base of ocelli clear (Fig. 7E, G).

Mouthparts: dorsal surface and anterior margin of labrum with long hair-like setae, dorsal surface with additional bristles, ventral surface with two oblique lines of hair-like setae; anterior margin almost straight, lateral margins slightly convex (Fig. 8A). Left mandible: apex of both inner and outer incisor divided into three denticles, outer incisor slightly thinner than inner one; prostheca composed with a distinct spur and a tuft of spines (Fig. 8B). Right mandible: outer and inner incisor divided into two denticles, prostheca composed with a tuft of spines, a line of hair-like setae on mesal margin near molar (Fig. 8C). Hypopharynx: lingua nearly circular, with hair-like setae on ventral apex; superlinguae with straight lateral margins, apex with hair-like setae too (Fig. 8D). Maxillary palpi yellowish brown, length ratio of segments I and II = 1.0: 2.0; apical segment covered with dense hair-like setae and slightly broader than basal one. Two gill tufts between maxilla and labium (Fig. 8E). Galea-lacinia of maxilla with two apical canines, mesal margin with a row of hair-like setae, one distinct spine among them. Crown of maxilla and outer 1/2 surface of both sides with hair-like setae, an additional row of short hair-like setae near canine on ventral surface (Fig. 8F). Glossae and paraglossae of labium heart-shaped, the latter broader than the former, surface of them with dense hair-like setae, those on margins longer. Labial palpi darker, basal segment: apical segment = 1.0: 2.0, outer margin with long hair-like setae, inner 1/2 surface of it with brush-like setae (Fig. 8G).

Thorax: color brown, with a median pale line and several irregular pale dots or markings on dorsal surface. Mesosternum and metasternum with a projection respectively directed posteriorly, the latter one broader and shorter (Fig. 7F). Gill tuft on forecoxa with pale body but gray filaments. Femora of foreleg with three pale bands on base, middle and apex respectively, tibiae with two pale bands on base and apex, apical 1/2 of tarsi pale, claw pale but with golden apex (Fig. 9A). Inner margin of foreleg with long hair-like setae but outer margin with bristles; apical spine of tibiae ~ 3/4 tarsi, it slightly bent. Claw with five or six denticles (Fig. 9B). Femora: tibiae: tarsi of foreleg = 1.0: 1.0: 0.6. Color pattern of midleg similar to foreleg, both outer and inner margins with spine-like setae only. Claw of midleg with eight or nine denticles (Fig. 9C, D). Femora: tibiae: tarsi of midleg = 1.0: 0.6: 0.4. Color and setae pattern of hind leg similar to midleg (Fig. 9E). Ventral cleft of hind femora usually with four spines (Fig. 9F). Femora: tibiae: tarsi of hind leg = 1.0: 0.5: 0.3.

Abdomen: brown, with a pale median line on terga I–VI, an additional pair of pale median oval dots beside line. Posterolateral angles of terga I–VII extended into blunt lobe while those of terga VIII and IX extended into spines (Fig. 7A–D). Gills I–VII similar in color and structure but larger progressively from anterior to posterior except dorsal gill lobes I–V without apical spines. Each gill lobe with three sclerotized ribs, two along margins and one nearly on middle of dorsal surface. Each gill with three purple dots, a bigger median one, a small apical one and the smallest anterolateral one. Front margin of each gill lobe slightly straighter than hind margin; ventral gill filament tuft with purple median 1/2, other parts pale (Fig. 10A–G). Sternite IX with distinct semi-circular shape cleft in both sexes (Fig. 10H, I). Anterior 1/2 of tergum X pale, posterior 1/2 brown (Fig. 10J).

Caudal filaments: mesal margin of 2/3 cerci and bilateral margins of terminal filaments with strong hair-like setae. Tail with pale band at 2/3 length (Fig. 7H).

**Figure 11.**
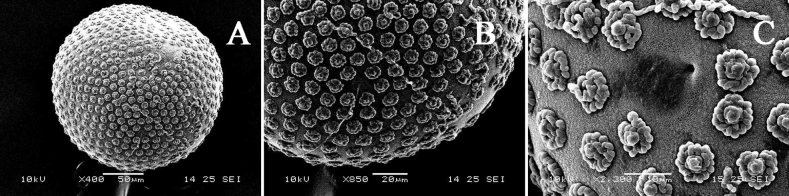
Egg of *I.kiangsinensis***A** overall view **B** surface enlarged **C** micropyle.

***Egg***. Spherical, densely covered with subequal tubercles, without clear reticulation (Fig. 11A, B); one micropyle observed (Fig. 11C).

#### Distribution.

China (Jiangxi, Fujian, Anhui, Zhejiang provinces).

#### Remarks.

The holotype and paratypes of this species were lost. Both the newly found specimens and original descriptions of Hsu (1936) show this is a valid species; therefore, a male from Jiangxi Province, China (same province as the types) is designated here as its neotype.

The nymphs of *I.kiangsinensis* have posterolateral projections on terga VIII and IX (Fig. 7A–D), which was missing in the description of You and Su (1987). However, Vasanth et al. (2019) and Muthukatturaja et al. (2021) reported wrongly that *I.kiangsinensis* has projections on terga I–IX.

### ﻿ Isonychia sexpetala

Taxon classificationAnimaliaEphemeropteraIsonychiidae

Tiunova et al., 2004

E0B5C9A1-98D3-588B-83E2-6CC922469BC5

Isonychia (Isonychia) sexpetala Tiunova et al., 2004: 10 (nymph and adults). Types from Russia and China; Zhou 2013: 196; Zhou et al. 2015: 245.

#### Material examined.

1 male imago, Nenjiang, Liuyuan, Qiqihar City, Heilongjiang Province, China, 1-VIII-2007, collected by Shilei Wang, Changfa Zhou.

#### Diagnosis.

**Figure 12.**
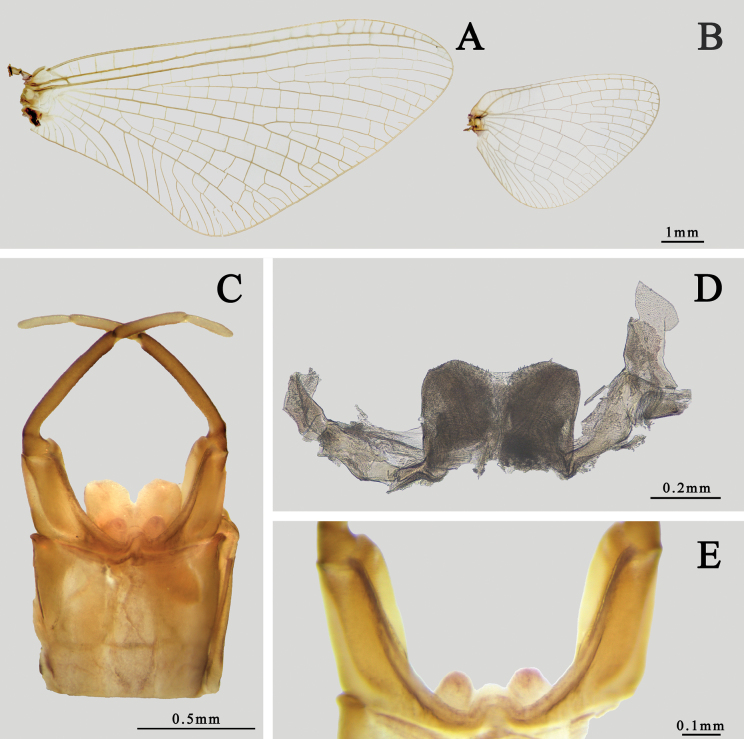
Male structures of *I.sexpetala***A** forewing **B** hindwing **C** genitalia (ventral view) **D** penes (dorsal view) **E** membranous processes (ventral view).

The male of this species is smaller than most congeners but similar to *I.ussurica*. It has no pigments on wings, MP of hindwings forked more apically than MA, but two forking points are close (Fig. 12A, B), segment II of forceps with straight inner margin, penes nearly cylindrical with convex distal margins and membranous processes with pigmented or sclerotized tip (Fig. 12C–E).

The males of *I.ivani* Tiunova et Gorovaya, 2010 and *I.sexpetala*Tiunova et al., 2004 are alike because they have similar cylindrical penes (Fig. 12D) and the basal 1/2 of their foretarsi is usually pale (Fig. 1D), but their membranous processes beneath penes are different. The processes of *I.sexpetala* have a rounded apex (Fig. 12E) while those of *I.ivani* are tapered. The nymphs of these two species can be separated by their color pattern of abdominal terga and gills: (1) the pale median longitudinal line of *I.sexpetala* is shorter than that of *I.ivani* (Tiunova et al. 2004; Tiunova and Gorovaya 2010), (2) gills of *I.ivani* have apical small dark dots while those of *I.sexpetala* are transparent.

#### Description.

See Tiunova et al. (2004).

#### Distribution.

China (Heilongjiang province); Russia (Tiunova et al. 2004).

### ﻿ Isonychia ussurica sibirica

Taxon classificationAnimaliaEphemeropteraIsonychiidae

Tiunova et al., 2004 (first record from China)

CC04373A-9999-5507-9F88-3F0F50ACB50F

Isonychia (Isonychia) ussurica sibirica Tiunova et al., 2004: 17 (nymph and adults). Types from Russian Siberia and Mongolia.

#### Material examined.

5 male imagos, Huma River, Huma County, Heilongjiang Province, China, 51°40.013′N, 126°36.590′E, 170 m, 17-VIII-2007, collected by Shilei Wang, Hui Xie; 3 nymphs, Heilongjiang, Arctic Village, Mohe County, Heilongjiang Province, China, 122°21.767′E, 53°28.499′N, 287 m, 14-VIII-2007, collected by Shilei Wang, Hui Xie; 3 male imagos, Nenjiang, Liuyuan, Qiqihar City, Heilongjiang Province, China, 123°57′E, 47°2′N, 1-VIII-2007, collected by Shilei Wang, Changfa Zhou; 1 male imago, Huma River, Tahe County, Heilongjiang Province, China, 52°18.273′N, 124°41.934′E, 358 m, 16-VIII-2007, collected by Shilei Wang, Hui Xie.

#### Diagnosis.

**Figure 13.**
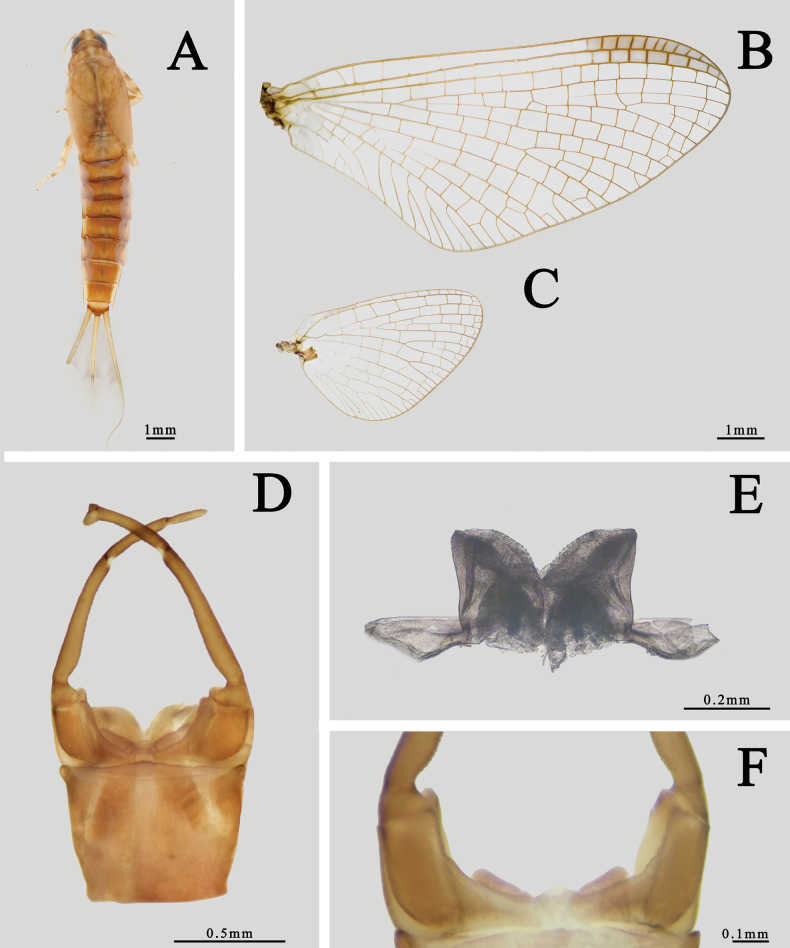
Imaginal and nymphal structures of *I.ussuricasibirica***A** nymph (dorsal view) **B** forewing of male **C** hindwing of male **D** male genitalia (ventral view) **E** penes (dorsal view) **F** membranous processes (ventral view).

The nymph of this species is smaller than others, abdomen without a clear pale line, tergum X with dark posterior half, and free margin of gills decorated with spines (Fig. 13A). The male of this subspecies is the same as *I.ussuricaussurica* (see below) except its forewing has no clear band (Fig. 13B–F).

#### Description.

See Tiunova et al. (2004).

#### Distribution.

China (Heilongjiang province), Russian Siberia, Mongolia (Tiunova et al. 2004).

### ﻿ Isonychia ussurica ussurica

Taxon classificationAnimaliaEphemeropteraIsonychiidae

Bajkova, 1970 (first record from China)

B3C2330E-0FD1-556A-BB18-FB4AB6E2F61B

Isonychia ussurica Bajkova, 1970: 148 (adults). Types from Khor River, Russia.
Isonychia sp. 1: Bajkova 1970: 153 (nymph) (named Isonychia (Isonychia) ussurica ussurica by Tiunova et al. 2004: 16 (male, female, and nymph)).

#### Material examined.

2 male imagos and 1 female imago, the river under No. 70 Bridge, Nancha County, Yichun City, Heilongjiang Province, China, 15–17-VII-2016, collected by Wei Zhang.

#### Diagnosis.

**Figure 14.**
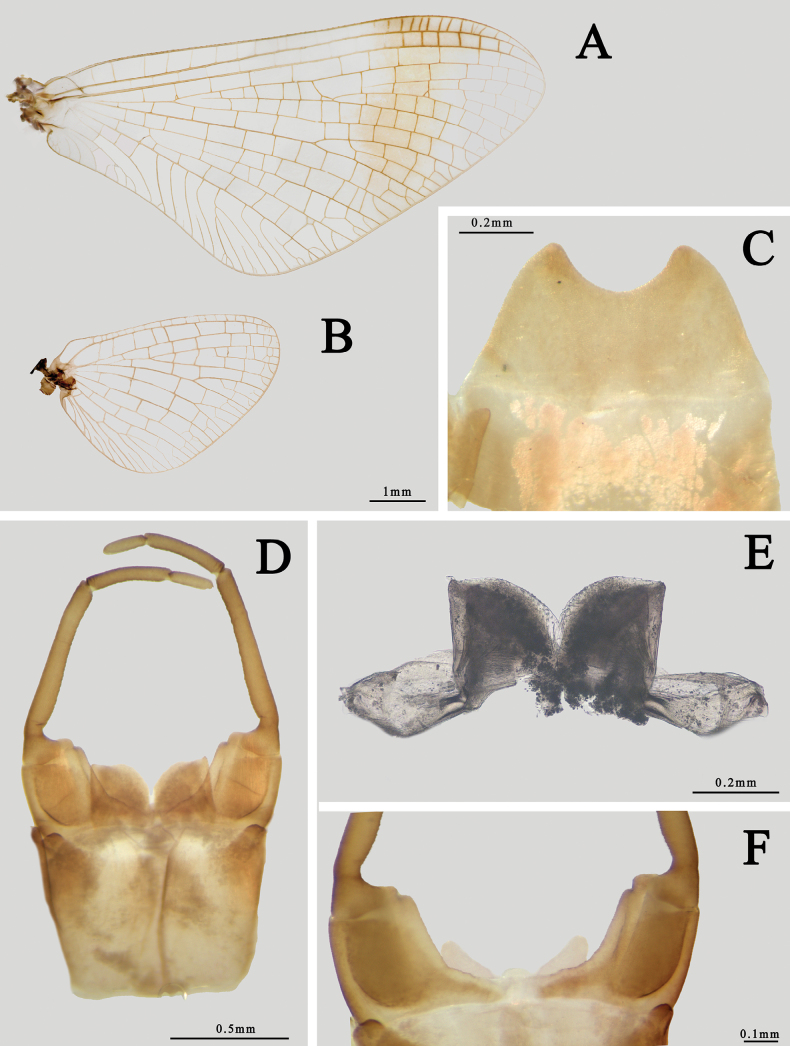
Imaginal structures of *I.ussuricaussurica***A** forewing of male **B** hindwing of male **C** subanal plate of female (ventral view) **D** male genitalia (ventral view) **E** penes (dorsal view) **F** membranous processes (ventral view).

The male of this subspecies can be identified by its body smaller than other species (~ 10.0 mm), forewing with pigmented band, MP and MA of hindwing forked equally (Fig. 14A, B), and nearly triangular penes and its membranous processes, slightly convex inner margin of forceps (Fig. 14D–F). The body of it is also usually uniformly reddish to reddish brown. Female size and color pattern are similar to males but without pigment on wings. Sternum IX has a narrowed posterior half and free margin is shallowly concave (Fig. 14C).

#### Description.

See Bajkova (1970) or Tiunova et al. (2004).

#### Distribution.

China (Heilongjiang province), Russia (Tiunova et al. 2004), Korea (Bae and Yoon 1997).

#### Remarks.

The size of brownish transverse band on forewing of our Chinese material seems smaller than that described by Bajkova (1970) and Tiunova et al. (2004) on Russian specimens (Fig. 14A). We regard this difference as a population variation.

Tiunova et al. (2004) stated clearly that two subspecies are distributed in different parts of Russia: *I.u.sibirica* in the western part such as Siberia and Mongolia while *I.u.ussurica* is found in the eastern part, the Russian Far East. Consistently, our collections of these two subspecies from Heilongjiang province show that the distribution of *I.u.sibirica* is more western than that of *I.u.ussurica*.

### ﻿ Isonychia vshivkovae vshivkovae

Taxon classificationAnimaliaEphemeropteraIsonychiidae

Tiunova et al., 2004 (first record from China)

FDE21473-7D8A-5AB6-9283-C36BA6969643

Isonychia (Isonychia) vshivkovae vshivkovae Tiunova et al., 2004: 24 (nymph and adults). Types from Russia.

#### Material examined.

23 male imagos, 3 female imagos, and 70 nymphs, Erdaobaihe Town, Fusong County, Jilin Province, China, 42°26.071′N, 128°06.882′E, 703 m, 23–26-VII-2008, collected by Shilei Wang, Guo Zhao; 30 nymphs, Songjianghe Town, Fusong County, Jilin Province, China, 42°10.568′N, 127°30.607′E, 685 m, 28-VII-2008, collected by Shilei Wang, Guo Zhao; 1 male subimago, Songhua River, Fusong County, Jilin Province, China, 42°19.591′N, 127°15.645′E, 423 m, 26-VII-2008, collected by Shilei Wang, Guo Zhao; 15 nymphs and 3 female imagos, Tou Dao Song Hua Jiang, Man Jiang Zhen, Fusong County, Baishan City, Jilin Province, China, 41.948515°N, 127.590697°E, 10-VIII-2022, collected by Xuhongyi Zheng; 3 male imagos and 3 female imagos, Toudao Observation Station, Toudaobaihe, Antu County, Jilin Province, China, 42°3809′N, 128°0244′E, 761 m, 23-VIII-2019, collected by Shuang Qiu, Zhengfei Li, Juanjuan Chen, Fengkun Cai; 9 female imagos and 2 female subimagos, National Highway 201, Sandaobaihe, Antu County, Jilin Province, China, 42°4677′N, 128°2014′E, 640 m, 25-VIII-2019, collected by Shuang Qiu, Zhengfei Li, Juanjuan Chen, Fengkun Cai.

#### Diagnosis.

**Figure 15.**
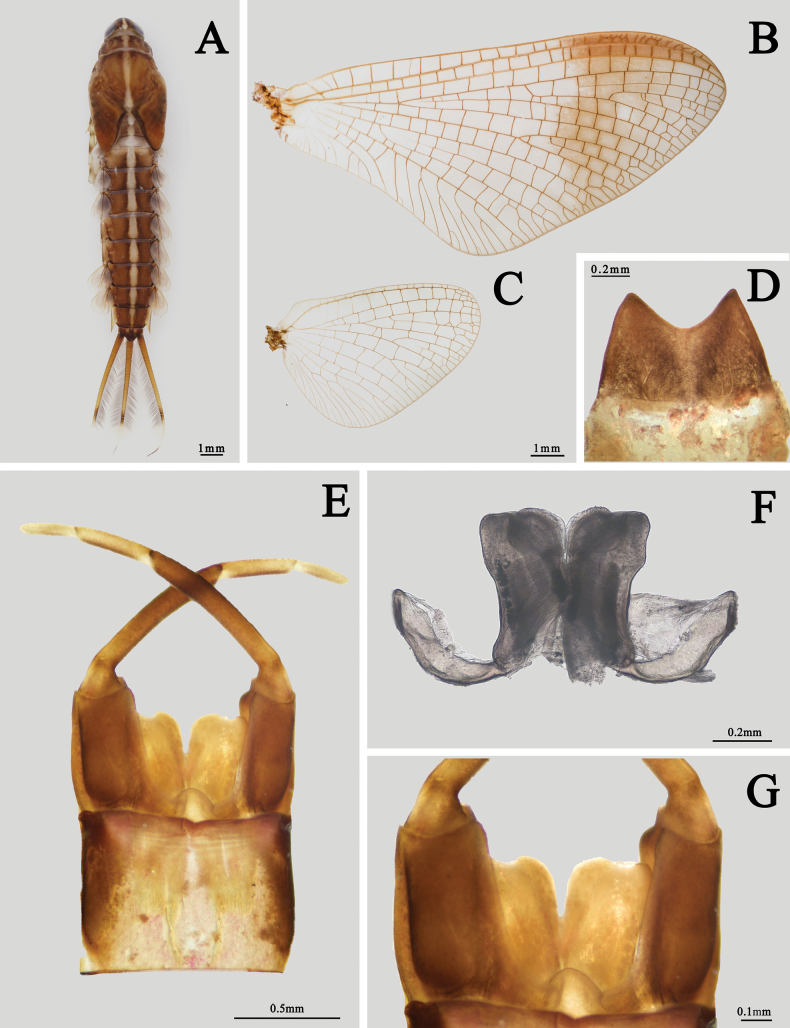
Imaginal and nymphal structure of *I.vshivkovaevshivkovae***A** nymph (dorsal view) **B** forewing of male **C** hindwing of male **D** subanal plate of female **E** male genitalia (ventral view) **F** penes (dorsal view) **G** male genitalia enlarged (ventral view).

The nymph of this species can be identified by its gills without spines along apical margins, body with a longitudinal median pale stripe from head to abdominal tergum IX, and tergum X with a pair of pale spots (Fig. 15A). The male imago can be differentiated easily by its outer half pigmented forewings, especially pterostigma, MP of hindwing forked equally as MA (Fig. 15B, C), relatively long and strong penes, apical half of penes slightly expanded and apical margins almost straight, membranous processes between penes and styligers are absent (Fig. 15E–G), basal half of each foretarsus is pale but apical half dark. Body is uniformly reddish brown to dark brown. Female imago has a similar color pattern of body and foreleg to male, pterostigma of forewing pigmented only, veins are clear. Posterior margin of sternum IX is shallowly concave (Fig. 15D).

#### Description.

See Tiunova et al. (2004).

#### Distribution.

China (Jilin province), Russian Far East (Tiunova et al. 2004).

#### Remarks.

Tiunova et al. (2004) divided *I.vshivkovae*Tiunova et al., 2004 into two subspecies, *I.v.vshivkovae* and *I.v.sinitshenkovae*. The former is distributed in the eastern part of Asian Russia and has pigmented forewings. Based upon the pigmented forewings and the collection location, our material is identified as *Isonychiavshivkovaevshivkovae*.

### ﻿Keys to *Isonychia* species from the Chinese mainland (modified from Tiunova et al. 2004)

Male imago

**Table:** 

1	Color of tarsal segments of foreleg entirely uniform dark brown (Fig. 2G)	***I.ignota***
–	Tarsal segments of foreleg dark in apical 1/2 and pale in basal 1/2 (Fig. 1D)	**2**
2	Inner margin of second segment of gonostylus distinctly convex (Fig. 13D)	**3**
–	Inner margin of second segment of gonostylus straight or weakly concave (Fig. 4C)	**4**
3	Forewing transparent (Fig. 13B)	***I.ussuricasibirica***
–	Forewing with pigmented band in apical 1/2 (Fig. 14A)	***I.ussuricaussurica***
4	Forewing with pigmented band in apical 1/2 (Fig. 15B)	***I.vshivkovaevshivkovae***
–	Forewing transparent (Fig. 4A)	**5**
5	Penis nearly triangular (Fig. 4E)	***I.kiangsinensis***
–	Penis nearly cylindrical (Fig. 1G)	**6**
6	Penis with a pointed angle (Fig. 1G)	***I.guixiensis***
–	Penis with a convex apical margin (Fig. 12D)	***I.sexpetala***

Nymph

**Table:** 

1	Apical margin of gills without spines, and abdominal tergum X with a pair of pale dots (Fig. 15A)	***I.vshivkovaevshivkovae***
–	Apical margin of gills (particularly gill lobes VI and VII) with spines, and abdominal tergum X with dark posterior 1/2 (Fig. 10A–G, J)	**2**
2	Abdominal terga I-IX with transverse pale spots (Fig. 13A)	***I.ussurica***
–	Abdominal terga I-IX with pale longitudinal midline (Fig. 7A)	**3**
3	Gill lobe with distinct purple to dark dot(s) (Fig. 10A–G)	**4**
–	Gill lobe colorless and transparent	***I.sexpetala***
4	Each gill lobe with a small apical dark dot (Fig. 2B)	***I.ignota***
–	Each gill lobe with three purple dots, a larger median one, a small apical one, and the smallest anterolateral one (Fig. 10A–G)	***I.kiangsinensis***

## ﻿Discussion

Tiunova et al. (2004) divided the eastern Palearctic *Isonychia* into two subgenera based on the shape of penes and styligers: Isonychia (Prionoides) Kondratieff & Voshell, 1983 and Isonychia (Isonychia) Eaton, 1871. All Chinese species in this paper belong to the latter.

Generally, species differences in both the nymph and adult stages are relatively few. The species can only be separated by tiny structures (the shape of penes and their membranous projections, the shape of forceps, and the spine pattern on the gill plate) or color patterns (stains on wings or pigment on forelegs) (Table 1).

**Table 1.** Comparison of *Isonychia* from Chinese mainland.

Stage	Character	Species						
		*I.guixiensis*	*I.ignota*	*I.kiangsinensis*	*I.sexpetala*	*I.ussuricasibirica*	*I.ussuricaussurica*	*I.vshivkovaevshivkovae*
male imago	coloration of wings	transparent	transparent	transparent	transparent	transparent	pigmented band	pigmented band
	shape of penis	nearly cylindrical with a pointed angle	nearly cylindrical with a convex apical margin	nearly triangular with a sharp apex	nearly cylindrical with a convex apical margin	nearly triangular	nearly triangular	relatively long with apical 1/2 of penis slightly expanded
	processes beneath penes	present	present	absent	present	present	present	absent
	2^nd^ segment of gonostyli	concave or straight	concave or straight	concave or straight	concave or straight	convex	convex	concave or straight
	foretarsal segments	dark brown in distal 1/2	entirely brown	dark brown in distal 1/2	dark brown in distal 1/2	dark brown in distal 1/2	dark brown in distal 1/2	dark brown in distal 1/2
nymph	apical margin of gills (particularly gill lobes VI and VII)	/	with spines	with spines	with spines	with spines	with spines	without spines
	abdominal tergum X	/	with dark posterior 1/2	with dark posterior 1/2	with dark posterior 1/2	with dark posterior 1/2	with dark posterior 1/2	with a pair of pale spots
	color pattern of abdominal terga I–IX	/	pale longitudinal midline	pale longitudinal midline	pale longitudinal midline	transverse pale spots	transverse pale spots	pale longitudinal midline
	color pattern of each gill lobe	/	one small apical dark dot	three distinct dots	transparent	transparent	transparent	transparent

**Figure 16.**
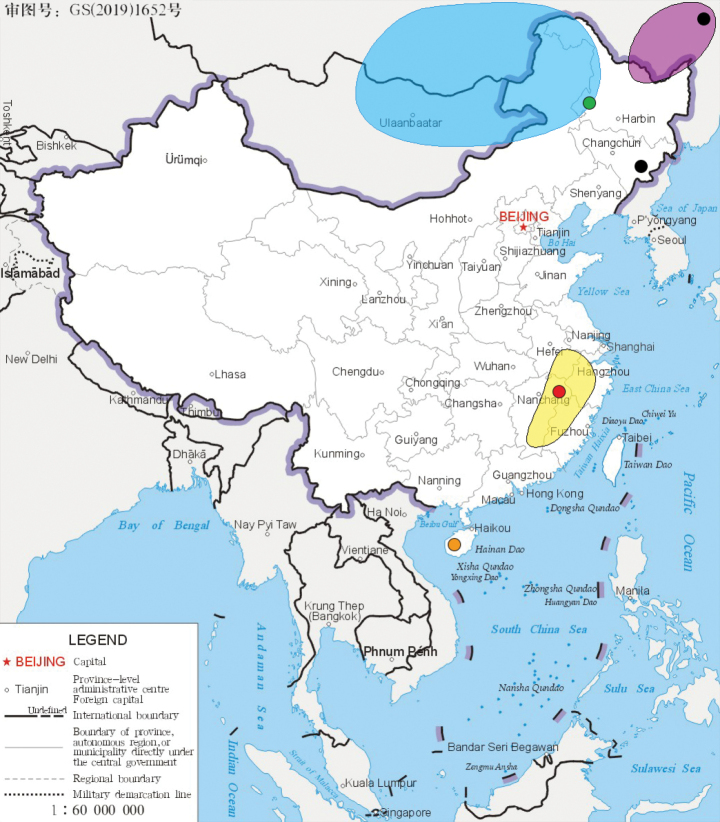
Distribution of *Isonychia* species from Chinese mainland (red represents *I.guixiensis*; orange represents *I.ignota*; yellow represents *I.kiangsinensis*; green represents *I.sexpetala*; blue represents *I.ussuricasibirica*; purple represents *I.ussuricaussurica*; black represents *I.vshivkovaevshivkovae*).

The six *Isonychia* species and one subspecies of Chinese mainland presented in the present paper show that, except *I.ignota* which has a wide distribution, the other five species can be divided geographically into southern and northern groups. The southern group includes two species (*I.guixiensis* and *I.kiangsinensis*) in south of the Yangtze River, while the northern one has three species and one subspecies (*I.sexpetala*, *I.ussuricasibirica*, *I.ussuricaussurica* and *I.vshivkovaevshivkovae*) found in northeastern China (Fig. 16). The huge gap in their geographical distribution highlights the need for more collections.

Additionally, Ulmer (1925) reported that *I.japonica* was found in southern Guangdong (Kuangtung) Province, China. However, Tiunova et al. (2004) clarified that this species is only found in Japan. In our opinion, this species may be found in northeastern China but will not be found in the southern part of this country. So for now, we exclude this species from Chinese mayfly fauna pending more material from Guangdong Province and further molecular work on the related species.

So far, at least seven *Isonychia* species and one subspecies have been confirmed as occurring in China (including *I.formosana*). Other material in our collection suggest that China has more species than are currently known and hosts the most diverse species in Asia.

## Supplementary Material

XML Treatment for Isonychia guixiensis

XML Treatment for Isonychia ignota

XML Treatment for Isonychia kiangsinensis

XML Treatment for Isonychia sexpetala

XML Treatment for Isonychia ussurica sibirica

XML Treatment for Isonychia ussurica ussurica

XML Treatment for Isonychia vshivkovae vshivkovae

## ﻿Additional information

### Author ORCIDs

Xin-He Qiang https://orcid.org/0000-0001-7991-1402

Chang-Fa Zhou https://orcid.org/0000-0001-8785-5228

### Data availability

All of the data that support the findings of this study are available in the main text.
